# The Effect of Cognates on Cognitive Control in Late Sequential Multilinguals: A Bilingual Advantage?

**DOI:** 10.3390/bs9030025

**Published:** 2019-03-08

**Authors:** Jorik Fidler, Katja Lochtman

**Affiliations:** Linguistics & Literary Studies, Vrije Universiteit Brussel, 1050 Brussels, Belgium

**Keywords:** multilingualism, bilingual advantage, Stroop task, cognates, orthographic neighbors, cognitive control, controlled language processing, German as a foreign language

## Abstract

The present study investigated the influence of Dutch-German cognates resp. orthographic neighbors on controlled language processing (i.e., response inhibition). Two monolingual Stroop tasks (Dutch and German) were performed by Dutch-speaking participants who could and could not speak German, and by French-speaking participants who could speak German. The question is whether or not cognate language processing affects cognitive control, resulting in a possible bilingual advantage. In the German Stroop task, we found additional advantages in congruent, as well as incongruent, trials for the two Dutch-speaking groups, which postulates the existence of a cognate resp. orthographic neighbor facilitation effect, even when participants only know one of the two cognate languages. The findings are discussed in relation to two possible factors that can modulate the effect of bilingualism on cognitive control: cognateness and orthographic neighborhood. The results suggest the existence of a notification mechanism in the bilingual brain. This mechanism would notify the bilingual brain when dealing with cognates and orthographic neighbors.

## 1. Introduction

In bilingual brains, lexical items of different languages are stored in the mental lexicon [1]. When late sequential bilinguals, also multilinguals who have acquired their L1 language system from birth on and their L2 language system during or after adolescence, learn a second language, their brain has to be aware of the fact that there is already a language stored in that brain. Therefore, the bilingual brain needs a certain control mechanism, not only to prevent it from between-language interference, but also to provide access to the right language during two-language processing [2]. This controlled language processing, which in the literature is often referred to as language control, takes place in a neural language control network, involving the prefrontal cortex (PFC), the anterior cingulate cortex (ACC), the inferior parietal cortex, and the caudate nuclei in the basal ganglia [3,4,5]. The function of this neural network not only involves language control, but also implicates cognitive control in other domains: the primary processes of this network are (response) inhibition, updating information in working memory, and shifting of mental sets [5,6].

Indeed, only when two or more languages are simultaneously accessible and activated is cognitive language control needed. The Language Mode Hypothesis [7], e.g., tries to point out when both languages are activated in the bilingual brain, and thus when language control is actually needed. The Language Mode Hypothesis assumes a continuum ranging from a purely monolingual context to a purely bilingual context. Any language context or communicative context, named ‘language mode’, can be seen as a point on this continuum. According to this theory, the L1 of a multilingual is always fully activated, but the more an actual language context approaches the bilingual endpoint, the more the L2 is activated and the more cognitive control the multilingual needs to avoid between-language interference. However, if the actual language context coincides with the monolingual endpoint, the L2 will not be activated at all and the multilingual will not need any cognitive control [7].

The Language Mode Hypothesis, and especially the deactivation of the L2 in the monolingual mode, has received much criticism from a connectionist distributed learning perspective. Further research has revealed that multilinguals cannot turn off their L2 in a monolingual context [1]. As a consequence, in bilingual brains, lexical items of all known languages are accessible and active, and are interacting with each other, even if only one language is being used at a given time point [8,9,10,11,12,13], both in language reception [14] and in language production [15]. That is why the bilingual brain constantly has to deal with a conflict situation: for each lexical item, it has to choose the right form, belonging to the right target language [8]. Bilingual language reception and production therefore requires the constant involvement of the cognitive control system.

Information about the way in which a bilingual brain saves and processes information is essential for understanding the question of whether and how multilinguals inhibit irrelevant information. More specifically, there needs to be clarity on the way that bilingual brains save and process two languages in one and the same brain. Following a first account of the connectionist Bilingual Interactive Activation (BIA) model [16], lexical items of the L1 and L2 are stored together in the bilingual brain. This would mean that every time the bilingual brain wants access to a word, all the words in the brain are activated, both the L1 words and the L2 words alike [16]. The BIA model consists of four levels: the lowest level is the feature level, followed by the letter level, the word level, and finally the language level or node. In order to be able to recognize a word, the bilingual brain should go through these four levels bottom-up (that is, from the lowest level to the highest level). Within this model, however, there are also top-down processes; the recognition of words is not only a bottom-up process, according to the BIA model, but also an interactive process in which both bottom-up and top-down processes take place. When a multilingual sees a word, such as the word ‘table’, the feature level gets activated: all features that match the letters of the word that needs to be recognized. The letter T, for example, consists of the features | and ‾. The activated features then form letters at the letter level. Letters that do not match the activated features are now suppressed. When all letters are formed, the word level forms words that match those letters. The word order of the letters is always respected at the word level: words that contain the same letters as the target word, but in a different order, are suppressed. For the example ‘table’, the letters T + A + B + L + E have a fixed order. In a bilingual brain, the word level contains two lexica: one lexicon for the L1 and one lexicon for the L2. However, those two lexica are stored together in the bilingual brain, so that every word that contains the right letter in the right order gets activated, independently of the language system the word belongs to. When all matching words have been activated, the language level activates the right language tag of the target word. This language tag or node then suppresses all activated words that do not match the target language [16].

The suppression takes place in the post-lexical phase: all words that match the activated letters, including the words of the non-target language, have already been activated in the bilingual brain. Only after this activation does the right language tag get activated, and only from then on, can the language tag suppress the previously activated words from the non-target language. However, a language tag cannot prevent words of the non-target language from being activated at the word level [16].

Evidence for the BIA comes from the Neighborhood Density effect (ND). This effect is based on orthographic neighbor words, words that only differ from each other in only one feature. Two visual neighbor words thus differ from each other in one letter, like ‘bee’ and ‘see’. According to the ND effect, the more orthographic neighbor words there are for a word, the longer it takes to recognize the word [17]. This effect also appears across different languages. The German word ‘Tee’ is also considered as a neighbor word of the English words ‘bee’ and ‘see’ [18]. “It has been shown that the recognition of a word belonging to L1, the active language, can be significantly affected by a large orthographic neighborhood in L2, the non-active language” [19] (p. 203). However, it is not clear whether this hypothesis still holds if there are two orthographically similar word forms with the same meaning.

The original BIA model only applies to the form recognition of individual words. That is why the BIA model was revised after a few years [20]. The BIA + model considers both the linguistic and the non-linguistic (i.e., semantic) context. The BIA model did not refer to the semantic context, whereas the BIA + model does. According to this model, the semantics of a word can thus give feedback to the orthographic word forms [21]. Words that agree both orthographically and semantically, such as cognates, would then, according to this model, be activated, and the semantic level would give semantic feedback to both orthographic word forms. Thus, if a cognate (e.g., the Dutch and German nouns *brief*/*Brief*) appears, two orthographic word forms (the Dutch and the German) are activated, and the semantic level gives feedback to both word forms, making the orthographic activation faster. According to the BIA+ model, cognates are then activated faster than non-cognates [22]. But what would this mean for the activation of orthographic neighbors with the same meaning (i.e., cognates)?

In order to answer these and similar questions, Jacquet and French (2002) introduced a further adaptation of the BIA+ model, which they refer to as BIA++. In this model, they suggest (according to the idea of a connectionist distributional learning network) the existence of unified multilingual lexicons, for which the existence of a language node is not needed [19]. We would like to hypothesize that according to such a model, orthographic neighborhood could have an effect, even when L2 learners have no knowledge of the target language in question, which would become apparent as a function of response inhibition control in a bilingual context, since this model encompasses a learning mechanism.

Controlled language processing in multilinguals could give them several cognitive advantages [23]. In the literature, these advantages are often referred to as the bilingual advantage. The bilingual advantage can be explained by the fact that multilinguals constantly need cognitive control in order to prevent the bilingual brain from between-language interference [8]. Several studies have shown that the bilingual brain is better trained in inhibiting irrelevant information compared to the monolingual brain [24] or less proficient bilingual brains [25]. More recent studies refer to the difference in the level of bilingualism to the bilingual advantage: the higher the level of bilingualism and the lower the age of acquisition of the L2, the higher the bilingual advantage [26,27]. However, some other studies did not find such a ‘bilingual advantage’ at all [28,29]. When looking at neuroimaging studies about this topic, it seems that bilingual brains are more efficient in dealing with interference: the brain network used by the monolingual brain is much bigger than the brain network used in the bilingual brain when dealing with interference, even if there is no difference in the behavioral level [30].

A good way of testing the efficacy of the bilingual cognitive control system is by running a Stroop task [31]. The Stroop task is a linguistic task measuring response inhibition control. In an original Stroop task, words are shown in a particular color. The words themselves can be color words or other nouns. Participants are asked to name the color in which the words are written. This task is easy when ‘neutral’ nouns are presented (‘control trials’, e.g., the word TABLE written in blue), and even easier when the color and the meaning of the word match (‘congruent trials’, e.g., the word BLUE written in blue). The task is much more difficult, however, when the color and the meaning of the presented word do not match (‘incongruent trials’, e.g., the word BLUE written in green).

Within response inhibition control, the Stroop task entails three effects: a facilitation effect, an interference effect, and a general Stroop effect. A facilitation effect occurs when the participant has to deal with a congruent trial: the time needed to name the color of the congruent trials is lower than the time needed to name the color of the control trials. The time needed to name the color of the incongruent trials, however, is longer than the time needed to name the color of the control trials. This effect is called the interference effect. An interference effect occurs because the automatic reading process and the color naming process are in conflict [32]. Finally, the overall Stroop effect is the sum of the facilitation effect and the interference effect, which is the time needed to name the color of the incongruent trials minus the time needed to name the color of the congruent trials. The overall Stroop effect is mostly used when a Stroop task only contains congruent and incongruent trials, but no control trials.

Considering both behavioral and neuroimaging studies about the bilingual advantage, it is clear that there is a difference in executive functioning between the monolingual and the bilingual brain, at least in tasks that involve interference suppression. Possible causal factors leading to that difference, however, remain much more speculative. In the current study, we investigated two factors that can modulate the effect of bilingualism on cognitive control: cognateness on the one hand, and orthographic neighborhood on the other hand.

Previous research has shown that cognates, which are defined as identical words with the same meaning in different languages [10], are processed faster by the bilingual brain than by the monolingual brain [33]. Cognates not only have an (almost) identical spelling in different languages, but are also identical on the phonological level, having the same meaning in those languages [10]. An example of Dutch-German cognates would be *nacht*/*Nacht* (night) or *vragen*/*fragen* (to ask). Because of the homologous meaning in different languages, cognates are processed significantly faster by bilingual brains: both items are supposed to be linked to the same semantic cue at the word level [33]. In fact, interlingual homographs, defined as words with an identical spelling and an identical phonology but with a different meaning in different languages, are processed significantly slower by the bilingual brain compared to the monolingual brain (i.e., the brain that does not know the target language in question). Because of the different meanings in the different languages, according to the BIA+ model, both items would be linked to different semantic cues in the mental lexicon of the bilingual brain [10]. Those differences between the monolingual brain and the bilingual brain would occur in all contexts, bilingual and monolingual language contexts alike [1]. Within bilingual brains, cognates are processed faster than non-cognates, both in a bilingual context and in a monolingual context [23]. Thus, the orthographic and semantic similarities are believed to have a facilitation effect on the bilingual brain [10,23]. In real life, however, most cognates have an almost identical, but no complete identical, orthography. The color words used in the present study also slightly differ in orthography. However, previous research affirms that those cognates follow the same tendency as completely identical cognates: the more identical the cognates, the bigger the facilitation effect [23].

What is less clear is the effect that cognates might have on multilinguals who only speak one of the cognate languages. In this case, the words cannot really be considered cognates. Instead, we speak of orthographic neighbors. The question then is, does the similarity between two different languages have a bilingual advantage in terms of lower interference and higher facilitation effects, even if multilinguals only speak one of the two similar languages? Such an advantage could only be explained from a BIA++ perspective, since this model incorporates the possibility of cognates and orthographic neighbor words being part of the same unified multilingual lexicon, resulting in “a distributed (i.e., non-localist) encoding” for the words in each (new) language [19] (p. 203). The advantage of this model is that it also includes a learning mechanism which is linked to this idea of distributed encoding. Word frequency is an important variable to be considered in distributed learning mechanisms, however. In this way, the BIA++ model is compatible with the Temporal Delay Hypothesis [20], which states that the more frequently a certain word is used, the faster it is believed to be activated. Therefore, in general, the activation of a word in the L1 would be faster than the activation of a word in the L2, because the L1 word is used more frequently than the (in the case of foreign language learners, sometimes yet to be learned) L2 word. As a consequence, cognates and orthographic neighbors with the same meaning could have an effect in an L2 Stroop task, because the L1 version of the cognate resp. orthographic neighbor is activated faster than the L2 version thereof. Previous research with primary school children with Dutch as L1 and English as L2 also found a beneficial cognate effect in the L2, but not in the L1 [34].

These issues could be dealt with by running a Stroop task in each of the cognate languages. On the phonological level, the similarity between two different languages has already been proven to have an effect on the bilingual brain: in a bilingual Stroop task (English and Japanese), the Stroop effects (i.e., the interference effect plus the facilitation effect) were bigger when the color words of both languages were phonologically similar [13]. However, English and Japanese have a different orthographic system. In another study, Dutch-English cognates were used in a Stroop task to test the possible effects that language similarity can have on cognitive control (i.e., response inhibition) [35]. The results of this study indeed showed a facilitation effect for the Dutch-English bilinguals, but the results were not compared to results of multilinguals who only spoke Dutch or English, combined with another language.

The question remains whether the orthographic similarity of two languages has an effect on multilinguals who do and do not speak both cognate languages and whether such an effect can be explained from the BIA++ model perspective of unified multilingual lexicons in foreign language learning in a distributed connectionist setting [19] (p. 203). In the current study, we investigated the results of L1 Dutch-L2 German multilinguals, L1 French-L2 German multilinguals, and the multilingual group L1 Dutch without knowledge of German, using a bilingual Stroop task with color words that are cognates in Dutch and German, but that are not cognates in French and Dutch. All participants are said to be multilinguals, because complete monolinguals hardly exist. The aim of the current study was to investigate whether the similarity between the Dutch and the German color words only had an effect on the Dutch-German multilinguals, or also on the multilingual group L1 Dutch without knowledge of German. Against the background described above, the current study will address the following research questions:

1. What influence, if any, do cognates have on the cognitive control in multilinguals?
2. What influence, if any, do orthographic neighbors have on the cognitive control in multilinguals?

These research questions can be supplemented with the following sub-question:

Do and to what extent do Dutch speaking learners of German experience a cognitive advantage (in terms of response inhibition) compared to

1. Dutch speaking students who have not yet learned German;
2. French speaking learners of German?

As for the Dutch-German multilinguals, we predict that the Dutch-German cognates will have an influence on the Stroop effects. The interference effect, on the one hand, would be bigger with cognates than with non-cognates, because both meanings of the cognates would not correspond with the color of the word. This double contradiction would lead to slower reaction times. The facilitation effect, on the other hand, would be bigger with cognates than with non-cognates, because both meanings of the cognates would correspond with the color of the word. This double confirmation would lead to faster reaction times. Taken together, because of the similarity between the color words in German and Dutch, the Stroop effects would be bigger in Dutch learners of German compared to the Stroop effects in French learners of German.

When comparing the general Stroop effects within the different groups, the Stroop effects should be bigger in the L1-Stroop task than in the L2-Stroop task, because of a higher interference effect and a higher facilitation effect in the L1 than in the L2. A Stroop effect will only occur if a participant understands the language the color words are written in. Dutch speaking participants who do not speak German and for whom the words are only orthographic neighbors to be learned, would therefore experience no Stroop effects in a German Stroop task. Any Stroop effects in the German Stroop task for these participants could only be explained through an orthographic neighborhood effect.

## 2. Materials and Methods

### 2.1. Participants

In total, 45 participants between the ages of 18 and 28 (*M* = 21.9, *SD* = 2.7) took part in the current experiment. The participants were divided into three groups: 15 L1 Dutch-L2 German multilinguals, 15 L1 French-L2 German multilinguals, and 15 multilinguals with L1 Dutch and without knowledge of German. All participants were university students from a Dutch-speaking or a French-speaking Belgian university. The Dutch-German multilinguals and French-German multilinguals were majors in German linguistics, whereas the multilingual group L1 Dutch without previous knowledge of German were psychology students.

All participants filled in a Language Background Questionnaire, in which they self-rated their (foreign) language proficiency. The average scores for each group can be found in Table 1, Table 2 and Table 3. All multilinguals with German as an L2 rated their L2-German proficiency 7 out of 10 or higher; all the multilinguals with L1 Dutch and without knowledge of German rated their L2-German proficiency below 2 out of 10. All participants had an L2-English proficiency of at least 6 out of 10. This means that all participants alike had completed English courses at high-school level (B1, of the CEFR) and can be considered as equal in this respect [36,37]. The Age of Acquisition (AoA) of L2-German of all learners of German was 17–18, because they only started learning German during the last year of high school or during the first year of university. This makes them late sequential multilinguals. The AoA for English is for all students alike: 13–14, that is, the second year of secondary education in Belgium. All participants signed an informed consent before taking part in the study.

### 2.2. Materials and Procedure

Two manual monolingual Stroop tasks were created using E-Prime 2.0 [38]: a monolingual L1 Stroop task (Dutch or French) and a monolingual L2 Stroop task (German). All color words used in the Stroop tasks were German-Dutch cognates, but none of them were German-French cognates. The color words used were *schwarz, gelb, grün, silber,* and *gold* (German); *zwart, geel, groen, zilver,* and *goud* (Dutch); and *noir, jaune, vert, argent,* and *or* (French). We used the Levenshtein distance as an index of similarity to control the color words in terms of their orthographic neihgbourhood across languages. The Levenshtein distance between two words is “the smallest number of substitutions, insertions, and deletions of letters required to transform one of the words to the other” [39] (p. 113). The Levensthein distance for the Dutch-German color words was always 2, except for *zwart/schwarz*. For this color word, the Levenshtein distance was 4, due to the German orthographic presentation for the sound /ʃ/. For the French-German color words, the Levenshtein distance was between 4 and 7 (the Levenshtein distances for the German-English color words were 0 for *gold/gold*, 1 for *silber/silver*, 2 for *grün/green*, 4 for *gelb/yellow*, and 6 for *Schwarz/black*). According to the Levenshtein index, the German-Dutch cognates can also be interpreted as orthographic neighbors for the participants who did not know any German. The control items used were *Baum, Stuhl, Prinz, Winkel,* and *Atem*; *boom, stoel, prins, hoek,* and *adem*; and *arbre, chaise, prince, angle,* and *souffle*. The word frequency of all control words was similar, both within and between the three languages. All items were presented in capital letters in size 60 Times New Roman font in the center of the screen, and could appear in either the Control (a control item in any color font), Congruent (i.e., the word BLUE presented in blue ink), or Incongruent (i.e. the word BLUE presented in green ink) condition. Both Stroop tasks consisted of 75 trials: 25 congruent trials, 25 incongruent trials, and 25 control trials. Prior to each Stroop task, there were 15 practice trials.

Each participant was sitting in a sound-attenuated room with a 15-inch computer screen to run the experiment. Participants were asked to press the color button that corresponded with the color of the ink the word was presented in. The task was explained in German by the teacher and was repeated on the computer screen. However, for the multilinguals without knowledge of German, the task was explained in Dutch. In order to give the participants the opportunity to ask questions, the experimenter stayed in the room during the practice trials, and then left the room.

Before each trial, a fixation cross appeared on the screen for 250 ms. After that, the trial appeared until the participant responded by pushing a button or until 4000 ms expired. Both reaction times and accuracy were collected and analyzed. Both Stroop tasks were run in a random order, and after each task, the participant had the opportunity to take a self-timed break. The whole experiment lasted about 15 min for each participant. For the analyses of the experiments, IBM SPSS Statistics version 25 [40] was used.

## 3. Results

### 3.1. General Results and Stroop Effects

#### 3.1.1. Experiment 1: Monolingual Stroop Task in L1

The analysis of the Stroop task was by means of calculating reaction times (RT) and accuracy rates. The mean RT’s of the correct trials for all subjects were calculated, and outlier RT’s beyond the range of 2.5 standard deviation of the mean were excluded from the process of analysis. After this trimming procedure, several Stroop effects indeed occurred in the reaction times. The overall Stroop effect (RT Incongruent trials—RT congruent trials) in the first experiment was significant for the French-German multilinguals in the French Stroop task (*t*(14) = 2.62, *p* = 0.02) (Table 4) and for the multilingual group L1 Dutch without knowledge of German in the Dutch Stroop task (*t*(14) = 2.33, *p* = 0.035) (Table 5), but not for the Dutch-German multilinguals in the Dutch Stroop task (*t*(14) = 2.09, *p* = 0.055) (Table 6). The same goes for the facilitation effect (RT neutral trials—RT congruent trials): it was significant for the French-German multilinguals in the French Stroop task (*t*(14) = 3.78, *p* = 0.002) (Table 4) and for the multilingual group L1 Dutch without knowledge of German in the Dutch Stroop task (*t*(14) = 2.35, *p* = 0.034) (Table 5), but not for the Dutch-German multilinguals in the Dutch Stroop task (*t*(14) = 1.16, *p* = 0.267) (Table 6). The interference effect was non-significant for all groups: for the French-German multilinguals (*t*(14) = 0.141, *p* = 0.89) (Table 4), for the Dutch-German multilinguals (*t*(14) = 1.15, *p* = 0.268) (Table 6), and for the multilingual group L1 Dutch without knowledge of German ( *t*(14) = 0.64, *p* = 0.53) (Table 5).

#### 3.1.2. Experiment 2: Monolingual Stroop Task in L2 German

The overall Stroop effect (RT Incongruent trials – RT congruent trials) in the second experiment was significant for the Dutch-German multilinguals (*t*(14) = 4.30, *p* = 0.0007) (Table 6) and for the multilingual group L1 Dutch without knowledge of German (*t*(14) = 4.40, *p* = 0.0006) (Table 5), but not for the French-German multilinguals (*t*(14) = 1.93, *p* = 0.074) (Table 4). The same goes for the facilitation effect (RT neutral trials—RT congruent trials): it was significant for the Dutch-German multilinguals (*t*(14) = 4.02, *p* = 0.001) (Table 6) and for the multilingual group L1 Dutch without knowledge of German (*t*(14) = 5.52, *p* = 0.00007) (Table 5), but not for the French-German multilinguals (*t*(14) = 1.50, *p* = 0.157) (Table 4). Similar to experiment 1, the interference effect was non-significant for all groups: for the Dutch-German multilinguals (*t*(14) = −0.89, *p* = 0.389) (Table 6), for the multilingual group L1 Dutch without knowledge of German (*t*(14) = −2.14, *p* = 0.0501) (Table 5), and for the French-German multilinguals (*t*(14) = 1.58, *p* = 0.136) (Table 4).

#### 3.1.3. Stroop Effects in French-German Multilinguals

In order to investigate the possible influence of Dutch-German cognates on the cognitive control of Dutch-German multilinguals, we need to compare the Stroop effects of the Dutch-German multilinguals with the Stroop effects of the French-German multilinguals who do not speak Dutch. Therefore, we needed to make the same comparisons with the French-German multilinguals as we did with the Dutch-German multilinguals. The comparison showed no significant differences. See Table 4.

#### 3.1.4. Stroop Effects in the Multilingual Group L1 Dutch without Knowledge of German

In order to know whether Dutch-German orthographic neighbors have an influence on the cognitive control of the multilingual group L1 Dutch without knowledge of German, we need to compare this group’s Stroop effects in the Dutch Stroop task with their Stroop effects in the German task. This allows us to see if the Stroop effects in both tasks differ significantly. When comparing the Stroop effects of the Dutch Stroop tasks with the Stroop effects of the German Stroop task, only the facilitation effect is significantly higher in the German Stroop task than in the Dutch Stroop task (*t*(14) = 2.35, *p* = 0.034). The overall Stroop effect and the interference effect did not significantly differ in this group. See Table 5.

#### 3.1.5. Stroop Effects in Dutch-German Multilinguals

In order to investigate the possible influence of Dutch-German cognates on the cognitive control of the Dutch-German multilinguals, we need to compare this group’s Stroop effects in the Dutch Stroop task with their Stroop effects in the German Stroop task. This comparison shows that only the facilitation effect is significantly higher in the German Stroop task than in the Dutch Stroop task (*t*(14) = 2.43, *p* = 0.029). The overall Stroop effect and the interference effect did not significantly differ in this group. See Table 6.

### 3.2. Language Effects

Possible language effects address the question of whether the presence or the absence of German as an L2 could have an effect on the Stroop effects of the multilinguals. With an independent *t*-test, the Stroop effects of multilinguals with knowledge of German as an L2 were compared with the Stroop effects of multilinguals without knowledge of German as an L2. The results showed that there were no significant differences in the Stroop effects of both groups.

### 3.3. Cognate and Orthographic Neighborhood Effects

Possible cognate and orthographic neighborhood effects address the question of whether the presence or the absence of cognates or orthographic neighbors could have an effect on the Stroop effects (i.e., interference and facilitation effects) of the multilinguals. With an independent *t*-test, the Stroop effects of multilinguals with Dutch as their L1 were compared with the Stroop effects of multilinguals with French as their L1. The results showed that in the German Stroop task, the multilinguals with French as their L1 experienced a significantly bigger interference effect compared to multilinguals with Dutch as their L1 (*t*(43) = −2.67, *p* = 0.01). All other Stroop effects did not significantly differ.

## 4. Discussion

### 4.1. Stroop Effects Within Groups

There were no significant differences between the different Stroop effects of both experiments. The Dutch-German multilinguals only experienced significantly higher facilitation effects in the German Stroop task than in the Dutch Stroop task, however. When looking at the Dutch-German and the multilingual group L1 Dutch without knowledge of German, the facilitation effect is also significantly higher in the German Stroop task compared to the facilitation effect in the Dutch Stroop task. The French-German multilinguals experience no difference in the facilitation effects of the French Stroop task and the German Stroop task. This means that a cognate resp. orthographic density effect appears in a multilingual (L2) context, even if multilinguals are not familiar with the cognate’s L2. These unexpected findings could point in the direction of the BIA++ model of a unified multilingual lexicon in a distributed connectionist setting of foreign language learning [19] (p. 203). Indeed, when assuming the presence of a learning mechanism, this ‘learning’ mainly takes place in the learner’s L2, i.e., German. Even the Dutch multilinguals who had no knowledge of German before the experiment seem to increasingly associate the German color words to the right color, reflected in the facilitation effect. This might mean that during the experiment, there is a learning process involved.

When looking at the interference effects, French-German multilinguals, as expected, experience no negative interference effect in the German Stroop task. Regarding the L1 Stroop task, no group experienced a negative interference effect. The Dutch-German multilinguals and the multilingual group L1 Dutch without knowledge of German, however, experienced a slightly negative interference effect (also a facilitation effect) in the German Stroop task. These results might be explained by the Neighborhood Density (ND) effect [17], stating that the more neighbor words a certain word has, the longer it takes to recognize that word. Additional research on this topic has found that this ND effect also occurs across languages. In the current research, this ND effect can slow down the recognition of color words in the German Stroop task for the Dutch-German multilinguals and the multilingual group L1 Dutch without knowledge of German, because in that task, both the L1 of the participants and German are activated in the participant’s brains: the cognate color words used in the current experiment are also orthographic neighbor words. Because of the slower semantic recognition of the color word due to the ND effect, the participant would already have responded to the color of the presented word, before even recognizing the meaning of the word. Therefore, the interference effect will disappear if the presented color word is a cognate and a neighbor word with the L1. Previous research already found that delayed word recognition in combination with enhanced cognitive control can reduce the Stroop effects in multilinguals [41]. In accordance with our predictions about the cognate influence of the multilingual group L1 Dutch without knowledge of German, this group would experience a similar effect, because the presented ‘new’ German color words are orthographic neighbor words of the familiar Dutch color words. In the L1 Stroop task, this ND effect does not seem to occur, however, because of a temporal delay. According to the Temporal Delay Hypothesis [20], the activation of a cognate word in L1 would be faster than the activation of the same cognate word in L2, because the L1 word is used more frequently than the L2 word. As a consequence, cognates can have an effect in an L2 Stroop task, because the L1 word is activated faster than the L2 word. In an L1 Stroop task, however, the participants might already have responded to the L1 word, before the activation of the L2 word takes place [34].

Note that some of the color words used in the Stroop task are also Dutch-English cognates and German-English cognates (grün/green, silber/silver, gold/gold) However, this did not influence the results of the experiment, because all participants were L2 English speakers with the same competence level (B1, CEFR). The French-German multilinguals therefore experienced the same effects in the German-English and Dutch-English cognate color words compared to the Dutch speaking groups. The similar results in this regard between the French- German and Dutch-German bilinguals are compatible with the BIA++ model; however, the English cognates and orthographic neighbor words being part of the same unified multilingual lexicon resulted in distributed (i.e., non-localist) encoding for the words in each language [19] (p. 203).

Despite general belief in the literature that all languages a multilingual knows are always activated and accessible in the bilingual brain [8,9,10,11,12,13], cognates and orthographic neighbors only seem to have a facilitation effect in an L2 context when running a Stroop task. Therefore, one could be seduced into the idea that in late sequential multilinguals, lexical items of the L1 and the L2 are stored separately in the brain, as found by, e.g., Kim et al. [42]. Nevertheless, it might still be the case that the lexical items of the L1 and L2 are stored together in the multilingual brain, but are affected by a temporal delay. Considering the Temporal Delay Hypothesis [20], the L2 variant of the cognate is activated slower than the L1 variant of the cognate, assuming that the L1 variant is used more frequently than the L2 variant [22], which, again, would be compatible with the BIA++ model on distributed connectionist learning, especially since the participants are still to be considered as foreign language learners.

It is highly likely, however, that a kind of notification mechanism is activated as soon as the bilingual brain is confronted with cognates or orthographic neighbors in the L2. This mechanism might be beneficial for the bilingual brain. However, note that in the current study, cognates and orthographic neighbors are only presented as separate words. Further research should be undertaken to investigate the influence of cognates and orthographic neighbors on the executive functioning of the bilingual brain, when those cognates/orthographic neighbors are presented in a broader syntactic and semantic context. Finally, in our Stroop experiment, we have opted for manual responses instead of vocal responses. However, these manual responses might also partly explain the lack of interference effects in some groups.

### 4.2. Stroop Effects Between Groups

Between group effects were analyzed in order to find possible cognate resp. orthographic neighborhood effects. These effects are believed to address the question of whether orthographic neighbors/the presence or the absence of German as an L2 could have an effect on the Stroop effects of multilinguals. However, no differences in orthographic neighborhood effects were found when comparing multilinguals with knowledge of German and multilinguals without knowledge of German. The facilitation effect of the Dutch speaking multilinguals in the German Stroop task was twice as high as the same facilitation effect of the French speaking multilinguals in the German Stroop task. This could also mean that the cognate effect is as high as the typical Stroop effect. The interference effect of the Dutch speaking multilinguals in the German Stroop task was significantly lower than the same interference effect of the French speaking multilinguals in the German Stroop task. These results confirm the theory that the interference effect disappears when dealing with cognates or orthographic neighbors (with the same meaning).

## 5. Conclusions

The purpose of the current study was to determine to what extent cognates and orthographic neighbors with the same meaning have an influence on bilingual controlled language processing. It turned out that both investigated factors (cognateness and orthographic neighborhood) seemed to modulate the effect of bilingualism on cognitive control to a certain extent. Cognates and neighbor words with the same meaning seem to have very similar positive or advantageous effects: leveling out to a large extent the interference effect on the one hand, and increasing the facilitation effect on the other hand. Based on these results, we would like to postulate the existence of a notification mechanism in the bilingual brain within a model of unified multilingual lexicons in a distributed connectionist setting of foreign language learning (BIA++) [19] (p. 203). This mechanism would notify the bilingual brain when dealing with cognates or orthographic neighbors with the same meaning. The precise nature of this mechanism remains to be elucidated. Although we would also like to assume the influence of a learning mechanism, this idea cannot be tested by using a Stroop-task. Further research, e.g., by using online research methods, might shed more light on the potential learning processes involved.

## Figures and Tables

**Table 1:** Self-rated proficiency scores for the Dutch-German multilingual group.

	Mean Score (Out of 10)	SD (Out of 10)
Dutch: writing (L1)	9.93	0.26
Dutch: speaking (L1)	9.93	0.26
Dutch: listening (L1)	9.93	0.26
Dutch: reading (L1)	9.93	0.26
German: writing (L2)	7.60	0.91
German: speaking (L2)	7.73	0.96
German: listening (L2)	8.47	0.74
German: reading (L2)	8.53	0.91
English: writing (L2)	7.70	0.98
English: speaking (L2)	7.87	1.13
English: listening (L2)	8.53	0.83
English: reading (L2)	8.67	0.82

**Table 2:** Self-rated proficiency scores for the French-German multilingual group.

	Mean Score (Out of 10)	SD (Out of 10)
French: writing (L1)	9.73	0.70
French: speaking (L1)	9.80	0.56
French: listening (L1)	9.93	0.26
French: reading (L1)	9.93	0.26
German: writing (L2)	7.33	0.82
German: speaking (L2)	7.07	0.26
German: listening (L2)	7.33	0.62
German: reading (L2)	7.53	0.83
English: writing (L2)	8.07	1.03
English: speaking (L2)	8.00	0.93
English: listening (L2)	8.60	0.91
English: reading (L2)	8.93	0.88

**Table 3:** Self-rated proficiency scores for the Dutch multilinguals without knowledge of German.

	Mean Score (Out of 10)	SD (Out of 10)
Dutch: writing (L1)	9.79	0.41
Dutch: speaking (L1)	9.93	0.26
Dutch: listening (L1)	9.79	0.56
Dutch: reading (L1)	9.86	0.35
German: writing	1.29	0.96
German: speaking	0.71	0.77
German: listening	1.36	1.06
German: reading	1.50	1.25
English: writing (L2)	7.21	0.99
English: speaking (L2)	7.36	1.22
English: listening (L2)	8.07	1.25
English: reading (L2)	7.71	1.18

**Table 4:** Stroop effects of the French-German multilinguals in the French Stroop task and in the German Stroop task.

	Mean Effect (ms)	SD (ms)
Stroop effect (French)	35.43	52.44
Stroop effect (German)	53.47	107.43
Facilitation effect (French)	33.60	34.45
Facilitation effect (German)	26.38	68.33
Interference effect (French)	1.83	50.30
Interference effect (German)	27.09	66.41

**Table 5:** Stroop effects of the multilingual group L1 Dutch without knowledge of German in the Dutch Stroop task and in the German Stroop task.

	Mean Effect (ms)	SD (ms)
Stroop effect (Dutch)	33.61	55.79
Stroop effect (German)	43.71	38.48
Facilitation effect (Dutch)	25.45	41.92
Facilitation effect (German)	61.00	42.81
Interference effect (Dutch)	8.16	49.37
Interference effect (German)	−17.30	31.25

**Table 6:** Stroop effects of the Dutch-German multilinguals in the Dutch Stroop task and in the German Stroop task.

	Mean Effect (ms)	SD (ms)
Stroop effect (Dutch)	24.16	44.72
Stroop effect (German)	42.26	38.09
Facilitation effect (Dutch)	10.44	34.99
Facilitation effect (German)	51.23	49.33
Interference effect (Dutch)	13.72	46.08
Interference effect (German)	−8.96	39.09
